# The progress to establish optimal animal models for the study of acute-on-chronic liver failure

**DOI:** 10.3389/fmed.2023.1087274

**Published:** 2023-02-09

**Authors:** Hengben Zhai, Jinming Zhang, Dabao Shang, Chuanwu Zhu, Xiaogang Xiang

**Affiliations:** ^1^Department of Infectious Diseases, Ruijin Hospital, Shanghai Jiao Tong University School of Medicine, Shanghai, China; ^2^Translational Lab of Liver Diseases, Department of Infectious Diseases, Ruijin Hospital, Shanghai Jiao Tong University School of Medicine, Shanghai, China; ^3^Department of Infectious Diseases, The Fifth People’s Hospital of Suzhou, Suzhou, China

**Keywords:** ACLF, animal model, hepatotoxic reagents, DAMPs, PAMPs

## Abstract

Acute-on-chronic liver failure (ACLF) defines a complicated and multifaceted syndrome characterized by acute liver dysfunction following an acute insult on the basis of chronic liver diseases. It is usually concurrent with bacterial infection and multi-organ failure resulting in high short-term mortality. Based on the cohort studies in ACLF worldwide, the clinical course of ACLF was demonstrated to comprise three major stages including chronic liver injury, acute hepatic/extrahepatic insult, and systemic inflammatory response caused by over-reactive immune system especially bacterial infection. However, due to the lack of optimal experimental animal models for ACLF, the progress of basic study on ACLF is limping. Though several experimental ACLF models were established, none of them can recapitulate and simulate the whole pathological process of ACLF patients. Recently, we have developed a novel mouse model for ACLF combining chronic liver injury [injection of carbon tetrachloride (CCl_4_) for 8 weeks], acute hepatic insult (injection of a double dose CCl_4_), and bacterial infection (intraperitoneal injection of *Klebsiella pneumoniae*), which could recapitulate the major clinical features of patients with ACLF worsened by bacterial infection.

## 1. Introduction

Acute-on-chronic liver failure (ACLF) is a clinical syndrome, defined by an acute hepatic/extrahepatic insult and subsequent rapid deterioration of liver function in patients with pre-existing chronic liver diseases or cirrhosis. This complicated syndrome is usually concurrent with bacterial infection and multi-organ failure resulting in high short-term mortality (1–4), and is becoming a major threat to those with chronic liver diseases (5).

Though the definitions and diagnostic criterion of ACLF vary worldwide, the main pre-existing chronic liver diseases are alcoholic liver disease (ALD) in the West and chronic hepatitis B (CHB) in the East, and the most common acute insults usually include excessive alcoholic consumption, hepatitis B virus (HBV) reactivation and drug-induced liver injury (DILI) (5). Bacterial infections are nearly inevitable events in ACLF patients according to the cohort with 1,343 consecutive patients from European Association for the Study of the Liver-Chronic Liver Failure (EASL-CLIF) Consortium revealing up to two-thirds detected (1, 6). Based on the cohort studies in ACLF worldwide (1, 7), the clinical course of ACLF could be divided into three major stages including chronic liver injury, acute hepatic/extrahepatic insult, and systemic inflammatory response caused by over-reactive immune system which worsened by bacterial infections.

Experimental animal model is pivotal for the study of ACLF (8). Several experimental ACLF models were established *via* combination of chronic and acute liver injury (9–13), including mice, rats or rabbits, however, none of them can recapitulate and simulate the whole pathological process of ACLF patients. Injection of carbon tetrachloride (CCl_4_) or bile duct ligation (BDL) surgery is the most commonly used way to mimic chronic liver injury in animal models, whereas injection of D-galactosamine (D-GalN) or lipopolysaccharide (LPS) is often used as acute injury. The combination of these chronic and acute liver injuries could lead to considerable mortality, but the mean survival period is too short after acute insult to applicate preclinical interventions. Moreover, bacterial infection could not be fully simulated *via* LPS injection and no viable bacterial infection is applicated in the above-mentioned models. This scenario surely hinders the investigations of mechanism research and drug screening in ACLF field.

Recently, we have developed a novel mouse model for ACLF combining chronic liver injury [injection of carbon tetrachloride (CCl_4_) for 8 weeks], acute hepatic insult (injection of a double dose CCl_4_), and bacterial infection [intraperitoneal injection of *Klebsiella pneumoniae* (*K.P.*)], which could recapitulate the major clinical features of patients with ACLF worsened by bacterial infection (14). This model could not only mimic the major three stages of ACLF, but also prolong the animal survival period with longer observation and intervention time for screening drugs and mechanism studies. In this review, the merits and demerits of emerging animal models are summarized, aiming to provide thoughts for researchers who focused on ACLF.

## 2. Current understanding of the mechanism of ACLF

The mechanism of ACLF is multifactorial and multifaceted (15). The most commonly underlying liver disease of ACLF is cirrhosis resulting from viral hepatitis or alcohol hepatitis. Progression of cirrhotic clinical course can be divided into three stages including pre-cirrhotic diseases, compensated cirrhosis and decompensated cirrhosis (16).

According to the triggers, ACLF can be categorized into two types. Clinical identifiable inducers include pathogen-associated molecular patterns (PAMPs) (4, 8, 17) such as bacterial components, and damage-associated molecular patterns (DAMPs) (18) such as pieces of necrotic or apoptotic cells. Besides, sepsis-induced ACLF also accounts for a large proportion, of which the most common are spontaneous bacterial peritonitis (SBP) (1) and severe alcoholic hepatitis (SAH), represents nearly 25% of ACLF cases (4, 19). Sepsis-induced ACLF is mainly caused by the dysfunctional immune response. According to the current reports about ACLF, there was an opinion that sepsis acting as an extrahepatic trigger, usually participated in the progression of ACLF (20). Except for those identifiable triggers, there are also some cases of ACLF with no obvious triggers that accounted for 40–50%. Till now there were three hypotheses that may account for this situation including the dysregulation of gut microbiota, translocation of PAMPs such as LPS, and DAMPs released by cell necrosis or apoptosis.

The proposition and confirmation of the systemic inflammation (SI) hypothesis in ACLF field is a big milestone for further understanding the mechanism of ACLF (21–23). In the pathophysiological mechanism of ACLF, systemic inflammation usually plays a pivotal role. ACLF patients with severe systemic inflammation, mostly accompanied with increased levels of pro-inflammatory cytokines, chemokines, growth factors, bioactive lipid mediators, such as IL-6, IL-8, and IL-1β (24). Excessive systemic inflammation will lead to “cytokine storm” in final, which is a critical factor causing immune-mediated tissue damage and organ injury (22, 23, 25–28). Systemic inflammation is mainly associated with PAMPs and DAMPs. Bacteria released PAMPs are recognized by pattern-recognition receptors (PRRs), and farther trigger the cascade amplification reaction. The most typical paradigm of these signaling pathways is LPS-Toll-like receptor 4 (TLR4), which contributes to the releasing of pro-inflammatory cytokines and type 1 interferons (IFNs). Apart from this, systemic inflammation can also occur in the absence of bacteria or virus infection, called sterile inflammation, mainly caused by DAMPs. DAMPs which expressed by broken cells, are also recognized by PRRs. Different forms of liver injury have different underlying mechanisms, respectively. Such severe systemic inflammation may result in several outcomes like tissue hypoperfusion, immune-mediated tissue damage and mitochondrial dysfunction (29). Among them, mitochondrial dysfunction serves a link in the progression of ACLF. There is a decreased oxidative phosphorylation and adenosine triphosphate (ATP) production in ACLF patients, which may exacerbate organ failures. Excessive pro-inflammation cytokines release consumes quantity of energy, combined with obstructed energy production, will finally result in immune paralysis (15). This suppression of immune system will increase the risk of secondary infection (30) and lead to higher mortality compared with those who remain free of immune suppression. MER tyrosine kinase (MERTK) also inhibits the immune system of ACLF patients (31). The number of MERTK expressing monocytes and macrophages is increased while the sensitivity toward LPS is decreased (31). Besides, Prostaglandin E2 (PGE2) and IL-10 also suppress immune system by reducing sensitivity of innate immune response and upregulation of regulatory immune cells (32, 33). It was also reported that the level of CD14^+^ monocytes and CD14^+^CD15^–^HLA-DR-myeloid-derived suppressor cells is higher in ACLF patients, which will suppress the immune response to bacterial PAMPs (31, 32).

## 3. Methods for inducing chronic or acute liver injury

According to current understanding of the clinical course and pathological mechanism of ACLF, the clinical course of ACLF could be divided into three major stages: chronic liver injury, acute hepatic/extrahepatic insult, and bacterial infection.

The principle of inducing liver fibrosis is the transformation of quiescent hepatic stellate cells (HSCs) to activated type expressing α-smooth muscle actin (α-SMA) and other extracellular matrixes. The first step to develop an animal model for ACLF is the induction of liver fibrosis/cirrhosis *via* some kinds of chronic liver injuries. Hepatotoxic chemical drugs induced liver injury and immune responses mediated liver injury are the most commonly used ways for chronic or acute liver damage (14). Hepatotoxic chemical drugs usually include CCl_4_, D-GalN, acetaminophen (APAP), concanavalin A (Con A), and thioacetamide (TAA). Heterologous serum or serum constituent, such as human serum albumin (HSA) and porcine serum (PS), are always used for immune responses mediated liver injury. In addition, surgical procedures induced liver injury is also adopted, such as common BDL surgery, hepatic ischemia/reperfusion and partial hepatectomy (HPx). The following summarizes the most recognized methods for inducing liver injury.

### 3.1. Carbon tetrachloride (CCl_4_)

Carbon tetrachloride is a powerful hepatotoxin which is used to induce liver fibrosis/cirrhosis through oral administration or injection (34, 35). CCl_4_ induced liver fibrosis can be reproduced in both rats and mice, even in rabbits and dogs. Liver injury caused by repeated injection of hepatoxic reagents, such as CCl_4_, would lead to the regeneration of hepatocytes, formation of fibrosis, and collapse of reticulin, and finally, result in liver architectural distortion and cirrhosis (34, 36). Besides, CCl_4_ is relevant with cell metabolism, the dysregulation of cations such as Ca^2+^, Na^+^, and K^+^ in cells and the activation of cytochrome 450 (CYP450), which also plays an important role in inducing liver steatosis (37). Single injection of CCl_4_ would result in acute hepatocytes damage and centrilobular necrosis (36, 38), which can be used to mimic acute hepatic insult for ACLF animal model. CCl_4_ can be given in several different routes including subcutaneous, intramuscular or intraperitoneal injections, oral administration and inhalation (39–43).

Carbon tetrachloride is the most commonly used reagent to induce acute liver injury and liver fibrosis due to its convenience and low cost. CCl_4_ induced liver fibrosis in mice can be developed in 6–8 weeks with continuous injection and is similar to clinical patients in pathophysiology. However, the hepatic fibrosis in mice induced by CCl_4_ is easily to reverse, and CCl_4_ would definitely cause damage to other organs. In addition, considering the toxicity and volatility of CCl_4_, this reagent should be carefully used in fume cupboard.

### 3.2. D-galactosamine (D-GalN)

D-Galactosamine is a powerful hepatotoxic reagent. Interfering with the uridine pool in the cell is the underlining mechanism of D-GalN in inducing liver injury. It induces lethal liver injury at large dose and would enhance the sensitivity of liver to LPS, an agonist of TLRs, playing synergetic liver damaging effects. Thus, D-GalN is widely used in combination with LPS in acute liver failure or endotoxemia animal models (44).

### 3.3. Thioacetamide (TAA)

Thioacetamide, an indirect hepatotoxin, exerts toxic effect *via* a two-step biotransformation mediated mainly by CYP450 2E1 to thioacetamide sulfoxide and further to thioacetamide sulfur dioxide (TASO_2_). TASO_2_, the dominating reactive metabolite of TAA, leads to hepatic cellular damage, apoptosis and necrosis *via* oxidative stress and downregulation of catabolism enzymes (45). TAA is applied to induce acute or chronic liver disease in experimental animal models (46). It is reported that the main features of clinical chronic liver disease, such as hepatic encephalopathy, metabolic acidosis, elevated transaminases, abnormal coagulopathy, and centrilobular necrosis, could be induced after TAA administration (47). However, the carcinogenicity of TAA to humans (class 2B rating) limits its extensive use.

### 3.4. Acetaminophen (APAP)

Acetaminophen N-acetyl-*p*-APAP, the most widely used antipyretic and analgesic drug, would cause severe liver injury even acute liver failure in the case of overdose in human (48). In mice, acute liver injury or failure can be induced following APAP overdose. Generally, at therapeutic dose, the majority of APAP will be metabolized in the liver to non-toxic metabolites (APAP-sulfate or APAP-glucuronide) and excreted *via* the bile and urine, whereas at toxic dose, the excess APAP will be oxidized in hepatocytes by CYP450 isoforms to highly toxic metabolite N-acetyl-*p*-benzoquinone imine (NAPQI) (49). The accumulation of NAPQI that causes hepatocellular necrosis and subsequent DAMPs secreted by damaged hepatocytes that activate innate inflammatory response eventually leads to acute liver injury/failure (50).

### 3.5. Concanavalin A (Con A)

Concanavalin A is a lectin isolated from Jack beans (also called Canavalia ensiformis). Lectins are proteins that bind to carbohydrates, and the specific binding structures for Con A are α-Mannose and α-Galactose structures found in sugars, glycoproteins and glycolipids (51). Con A is a well-known T cell mitogen that can activate the immune system, recruit lymphocytes and elicit cytokine production (52). Unlike the hepatoxic reagents, Con A induced acute liver injury in mice is mainly based on the activation of CD4 + T cells and the subsequent secretion of proinflammatory cytokines, mainly IFN-γ and TNF. The mouse model of Con A induced liver injury is commonly adopted for investigating the mechanisms of autoimmune hepatitis (AIH) (53).

### 3.6. Human serum albumin (HSA)

Human serum albumin, the most abundant serum protein in blood with a half-life of 19 days in humans (54), is a typical constituent of heterologous serum for murine. HSA is often used to develop the immunologic reaction induced chronic liver injury models in rats and mice (13, 55, 56). Immune mediated chronic liver injury induced by repeated administration of HSA would lead to typical liver fibrosis in mice or cirrhosis in rats. Subsequently, D-GalN plus LPS are administrated to establish ACLF model (56, 57). However, it is reported that the mortality of HSA administration during chronic liver injury or fibrosis-induction period is relatively high at 23% (56). The high mortality limits the application of HSA in establishing chronic liver fibrosis models.

### 3.7. Porcine serum (PS)

Immune-mediated hepatic injury models are easily developed *via* the administration of heterologous serum constituent such as HSA. But the high mortality during the period of HSA induced chronic liver injury in murine models impels the usage of other kinds of heterologous serum. Porcine serum has been used to induce hepatic fibrosis for a long time, but the mechanism is uncertain until 1996. In order to investigate whether the hepatic fibrosis is caused by immune responses, Bhunchet et al. (58) divided rats into two groups, the porcine serum tolerant group and control group. Rats in the tolerant group had been injected with porcine serum peritoneally from the day of birth for 18 weeks while 8 weeks old rats in the control group received porcine serum injection for 10 weeks peritoneally. And antibody against porcine albumin level in the tolerant group is extremely lower than the control group, which suggests that no immune responses exist in tolerant group. Besides, no rats in the tolerant groups developed hepatic fibrosis. Based on this study, the mechanism of porcine serum induced hepatic fibrosis can be verified. Porcine serum is a suitable candidate for inducing the immune mediated liver injury models because of the low mortality reported (9). Compared with CCl_4_, immune metabolism disorder is the basis of PS induced liver fibrosis, which mainly used to mimic the chronic liver injury caused by HBV infection or autoimmune liver diseases mediated cirrhosis (59–62). From histological perspective, the infiltration of monocytes and the formation of fibrosis around portal vein are the remarkable features of this model (60). PS induced immune mediated chronic liver cirrhosis demonstrates great popularity due to its economic efficiency and practicability.

### 3.8. Bile duct ligation (BDL)

Bile duct ligation is a typical surgical approach established since 1930s to simulate extrahepatic biliary obstruction that leads to biliary cirrhosis in rats or mice (63–65). The core procedure for BDL surgery is that rats or mice are subjected to double ligation of the common bile duct with section between the two ligatures, then hyperbilirubinemia would be mimicked in these BDL rats (64, 65). In BDL models, acute obstructive jaundice occurs and the expression of pro-inflammatory cytokines (such as TNF, IL-6, and IL-17) and pro-fibrotic proteins (such as collagen-α1, MMP-2, and TIMP-1) are induced in portal areas, which would progress to cirrhosis (66–68). Though liver inflammation and fibrosis are well displayed in the BDL models, the surgical procedures are difficult to handle that limits its wide application.

## 4. Methods for mimicking bacterial infection

### 4.1. Bacterial component: Lipopolysaccharide (LPS)

Lipopolysaccharide is the main component of the outer membrane of all Gram-negative bacteria, which is mainly consist of three parts, the lipid A (or endotoxin), a core phosphorylated oligosaccharide, and a variable specific long polysaccharide chain composed of repeating oligosaccharide (or O-antigen) (69, 70). LPS, one of the classical PAMPs, is a powerful mediator of systemic inflammation and septic shock *via* activating the PRRs-TLR4/TLR2 signaling pathways (4, 71–73). Normally, LPS first binds to LPS-binding protein to form an activated receptor complex with myeloid differentiation factor 2 (MD2), the CD14, and TLR4. Signals are transduced to intracellular proteins (MyD88, IRAKs, TRAFs, and NIK) by the activated receptor complex, generating an intricate network of cellular responses, activation of the NF-kB pathway, and secretion of a large amount of pro-inflammatory cytokines (74). Usually, LPS is co-administrated with D-GalN to induce acute liver injury models or fibrosis models in rats or mice which has been widely used and extensively studied (75, 76).

### 4.2. Polymicrobial infection: Cecal ligation and puncture (CLP)

In order to investigate sepsis and sepsis-associated multiorgan failure, several experimental animal models with polymicrobial infection have been established to mimic the pathophysiological changes in septic patients (77). Cecal ligation and puncture (CLP) in murine is the most widely used and typical model for experimental sepsis which has been developed more than 30 years. Moreover, the CLP model is considered to be an ideal model for the induction of polymicrobial sepsis (77, 78). The surgical procedure features of CLP include midline laparotomy, ligation below the ileocecal valve, and needle puncture of the cecum (79). The severity of CLP model can be tailored via the ligation length of cecum and the needle puncture size.

### 4.3. Polymicrobial infection: Cecal slurry (CS)

Since the major problem for CLP-based polymicrobial sepsis model is consistency of the surgery, cecal slurry (CS) injection based polymicrobial peritoneal sepsis model is developed to solve the consistency problem and simplify the surgical procedure (80). CS-induced sepsis model is an infectious model with bacterial colonization, systemic inflammation and dose-dependent mortality without surgery, which is widely accepted and now considered as the “gold standard” model for murine neonatal sepsis study (80, 81). The advantages of CS-induced polymicrobial sepsis are no surgical procedures, a single CS donor can be administrated in a large number of animals, and easy to perform.

### 4.4. Single bacterial infection: *Klebsiella pneumoniae* (*K.P.*)/*Escherichia coli* (*E. Coli*)/*Salmonella typhimurium* (*S. Typhimurium*)

To study the role of liver during bacterial infection in different organs, several experimental bacterial animal models have been developed. For systemic single bacterial infection model, mice or rats are injected intraperitoneally with a certain dose [colony-forming unites, (CFU)] of *K.P.* or *Escherichia coli* (*E. Coli*) or *Salmonella typhimurium* (*S. Typhimurium*) directly (82–84). For the lung bacterial infection model, animals are given *K.P.* through a non-invasive intratracheal intubation (85).

## 5. Existing experimental animal models for ACLF

**FIGURE 1:**
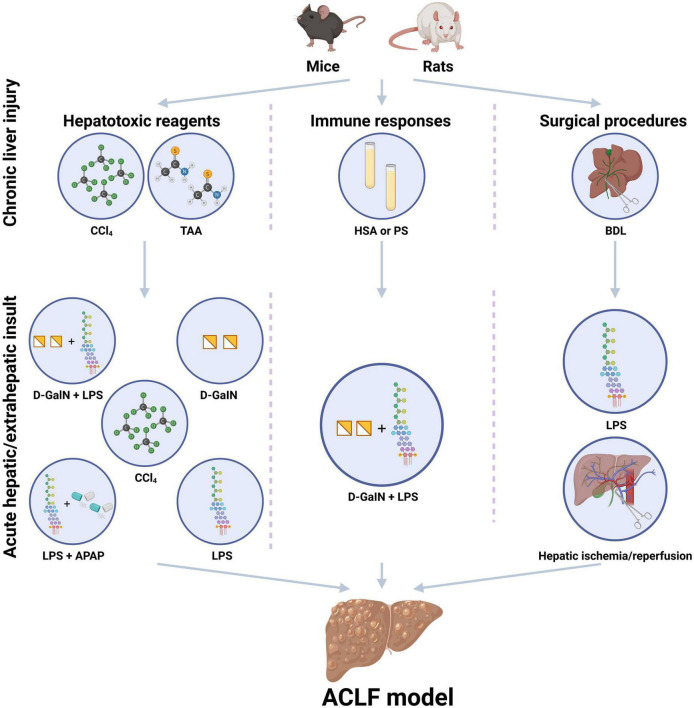
Existing experimental animal models for the study of acute-on-chronic liver failure (ACLF). The existing experimental animal models for ACLF usually contain the steps of chronic liver injury and acute hepatic/extrahepatic insult and could be classified into three major patterns, including ACLF models induced by hepatotoxic reagents, immune responses, or surgical procedures. Hepatotoxic reagents usually include carbon tetrachloride (CCl_4_), D-galactosamine (D-GalN), acetaminophen (APAP), thioacetamide (TAA) concanavalin A (Con A), and lipopolysaccharide (LPS). Immune responses induced ACLF models are usually based on heterologous serum or serum constituent, such as human serum albumin (HSA) and porcine serum (PS). Surgical procedures induced liver injury includes common bile duct ligation (BDL) surgery, partial hepatectomy (HPx) and hepatic ischemia/reperfusion. D-GalN and LPS are always used as acute insults. CCl_4_, carbon tetrachloride; PS, porcine serum; HSA, human serum albumin; BDL, bile duct ligation; TAA, thioacetamide administration; LPS, lipopolysaccharide; D-GalN, D-galactosamine; APAP, acetaminophen. (Created with BioRender.com).

Currently, the existing experimental animal models for ACLF could be classified into three major types, including ACLF models induced by hepatotoxic reagents, immune responses, or surgical procedures respectively, (Figure 1), which are created *via* the combination of the above-mentioned methods sequentially to simulate the pathogenic course of this devastating disease. The following displays the principles and methods used in the existing experimental animal models for ACLF (Table 1).

**TABLE 1:** The animal models used for the study of acute-on-chronic liver failure (ACLF).

Animal	Chronic liver injury/Fibrosis	Acute liver injury/Insult	Bacterial infection	Mortality during chronic injury	Mean survival time after acute insult	References
Wistar	20% HSA to induce liver injury/Fibrosis. First subcutaneous injection of HSA 4 mg for 24 days. Second intravenous injection of HSA 2.5–4 mg for 2 months	D-GalN 400 mg/kg. LPS 100 μg/kg. Injected intraperitoneally.	None	20–30%	16.1 ± 3.7 h. Less than 1 day	Liu et al. (102)
Sprague–Dawley	Dissolution of CCl_4_ in peanut oil (volume, 1: 1). Injection of CCl_4_ at a dose of 1.5 mL/kg in the 1st month and 2.0 mL/kg in the 2nd month once every 3 days. Injected intraperitoneally	D-GalN 500 mg/kg. LPS 80 μg/kg. Injected intraperitoneally.	None	11.25%	Less than 1 day	Ni et al. (95)
Sprague–Dawley	Dissolution of CCl_4_ in peanut oil (10%). Doses are modified according to liver function and body weight of rats. Injected intraperitoneally	D-GalN 700 mg/kg. Injected intraperitoneally	None	Not described	2–3 days	Zhang et al. (86)
Wistar	CCl_4_ inhalation 3 times a week for 15–16 weeks and received phenobarbital (0.3 g/l) in drinking water	LPS 1 mg/kg. Injected intraperitoneally	None	Not described	Less than 1 day	Tripathi et al. (12)
Wistar	Injection of porcine serum at a dose of 0.5 mL twice per week for 11 weeks. Injected intraperitoneally	LPS 50 μg/kg. Injected intravenously. D-GalN 600 mg/kg. Injected intraperitoneally	None	Not described	Less than 1 day	Li et al. (9)
Sprague–Dawley	BDL	LPS 1 mg/kg. Injected intraperitoneally	None	10–20%	Not described	Shah et al. (113) Balasubramaniyan et al. (11)
Wistar	BDL	Hepatic ischemia/Reperfusion	None	Not described	Not described	Hu et al. (106)
Sprague-Dawley	Dissolution of TAA in saline (250 mg/kg). Injected intraperitoneally. Twice a week, for 10 weeks	LPS 1 mg/kg Injected intraperitoneally or intravenously	None	Not described	Less than 1 day	Tripathi et al. (12)
BALB/c	Dissolution of CCl_4_ dissolved in mineral oil (20%). Injection of CCl_4_ at a dose of 2 uL/g. Injected intraperitoneally twice a week for 6 weeks	D-G alN 500 μg/g. LPS 10 ng/g. Injected intraperitoneally	None	Not described	Not described	Bai et al. (94)
C57BL/6	Dissolution of CCl_4_ in olive oil. Injection of CCl_4_ at a dose of 0.5 mL/kg. Gavage twice weekly for 6 weeks	LPS 4 mg/kg. Injected intraperitoneally	None	Not described	Not described	Engelmann et al. (91)
C57BL/6	Dissolution of CCl_4_ in olive oil. Injection of CCl_4_ at a dose of 0.5 mL/kg. Gavage twice weekly for 6 weeks	GalN 1,000 mg/kg. Injected intraperitoneally	None	Not described	Not described	Kondo et al. (92)
C57BL/6	Dissolution of CCl_4_ in olive oil. Injection of CCl_4_ at a dose of 0.1 mL/kg for first 3 weeks; 0.2 mL/kg for next 3 weeks; 0.5 mL/kg for last 4 weeks. Injected intraperitoneally twice a week for 10 weeks	LPS (50 μg/kg) + APAP (350 mg/kg). Injected intraperitoneally	None	Not described	Not described	Nautiyal et al. (98)
BALB/c	Dissolution of CCl_4_ in olive oil (10%) 10% CCl_4_ (5 mL/kg) twice a week for 8 weeks. Injected intraperitoneally	50% CCl_4_ 4 mL/kg. Injected intraperitoneally	None	Not described	Not described	Zhang et al. (93)
C57BL/6J	Dissolution of CCl_4_ in olive oil (volume, 1: 9). 0.2 mL/kg twice a week for 8 weeks. Injected intraperitoneally	CCl_4_ 0.4 mL/kg on the 1st day of week 9. *Klebsiella pneumonia* 1,000 CFU/mouse. Injected intraperitoneally	Yes	None	3–5 days	Xiang et al. (14)
C57BL/6J	Dissolution of CCl_4_ in olive oil (volume, 1: 9). Injection of CCl_4_ at a dose of 0.2 mL/kg twice a week for 8 weeks. Injected intraperitoneally.	CCl_4_ 0.4 mL/kg on the 1st day of week 9. Injected intraperitoneally. Cecal ligation and puncture (CLP) surgery on the 2nd day.	Yes	None	3–5 days	Xiang et al. (14)
C57BL/6J	Dissolution of CCl_4_ in olive oil (volume, 1: 9). 0.2 mL/kg twice a week for 8 weeks. Injected intraperitoneally.	CCl_4_ 0.4 mL/kg on the 1st day of week 9. *Salmonella typhimurium* 8,000 CFU/mouse. Injected intraperitoneally.	Yes	None	3–5 days	Zhang et al. (84)

### 5.1. Hepatotoxic reagents induced ACLF models

Hepatotoxic reagents induced ACLF models are the most commonly used models and suitable for mimicking most clinical cases.

#### 5.1.1. CCl_4_ + D-GalN/LPS

The combination of repeated CCl_4_ administration and subsequent D-GalN/LPS could perfectly mimic the chronic liver injury and acute insult of ACLF. Repeated treatments of CCl_4_ result in chronic liver injury which would lead to fibrosis in mice or cirrhosis in rats. Moreover, the systemic inflammatory response caused by bacterial infection are also simulated by LPS, one of the typical PAMPs secreted by Gram-negative bacteria.

As illustrated in Table 1, CCl_4_ is the most frequently used method to establish ACLF models. Normally, rats are selected to administrate CCl_4_ *via* intraperitoneal injection, subcutaneous injection, intragastric gavage or inhalation for 6–8 or 8–12 weeks to induce the chronic liver injury with a fibrotic or cirrhotic state (12, 86–88), then D-GalN alone or D-GalN plus LPS are administrated (86, 89). Meanwhile, ACLF models can also be established in mice treated with CCl_4_ for 6–8 weeks to a fibrotic state then following the D-GalN/LPS administration (90–94).

Ni et al. (95) dissolved CCl_4_ in peanut oil [(volume, 1: 1) 1.5 ml/kg in the first month, 2.0 ml/kg weight in the second month], and further injected with LPS (80 μg/kg) and D-GalN (500 mg/kg) to induce ACLF, aiming to illustrate the mechanism of degradation of regulatory T cells. Tripathi et al. (12) summarized three ACLF models including BDL, CCl_4_, and TAA induced liver cirrhosis, respectively, to verify the protective efficacy of Simvastatin. In this study, CCl_4_ group received CCl_4_ inhalation 3 times weekly for 10 weeks combined with phenobarbital in drinking water (0.3 g/L) in order to short the period to form liver cirrhosis.

The combination of CCl_4_ and D-GalN/LPS for inducing ACLF models is easily to perform and suitable for mimicking most clinical cases. The key defect of these models is that the mean survival periods after treating with D-GalN/LPS are too short to conduct preclinical interventional studies.

#### 5.1.2. TAA + LPS

It is reported that chronic liver injury induced by TAA in rats can lead to cirrhosis with typical features such as hepatic encephalopathy, abnormal coagulopathy and centrilobular necrosis (46, 47). Tripathi et al. (12) developed three chronic liver injury models in rats through CCl_4_ inhalation, BDL, and TAA administration, respectively, followed by intraperitoneal or intravenous administration of LPS to mimic ACLF. It was confirmed that LPS administration in these cirrhotic rats could recapitulate the features of ACLF syndrome in some extent. For the TAA model, male Sprague–Dawley (SD) rats were treated intraperitoneally with TAA (250 mg/kg) twice a week for 10 weeks (96, 97) and then treated with LPS (1 mg/kg) to develop ACLF model (12). Though the combination of TAA and LPS is also easy to perform, reports about TAA plus LPS induced ACLF model are rare and details of this model need further studies to display and elucidate.

#### 5.1.3. CCl_4_ + APAP/LPS

It is theoretically possible that co-administration of APAP and LPS in CCl_4_ induced chronic liver injury mice would develop a kind of experimental ACLF model. However, there is only one group has reported the establishment of ACLF model in this kind until 2021 (98). Nautiyal et al. (98) have confirmed that APAP plus LPS can be served as a hepatic insult for constructing ACLF model. In their study, mice were intraperitoneally administrated of CCl_4_ (0.1–0.5 ml/kg) twice a week for 10 weeks, followed by APAP (350 mg/kg) and LPS (50 μg/kg) injection intraperitoneally (98). It is reported that progressive hepatocyte necrosis, liver failure, impaired regeneration, development of portal hypertension and multi-organ dysfunction were demonstrated in this new ACLF model after 11 days (98). This model showed a prolonged survival period after hepatic insult, which would surely provide us a choice to carry out interventional studies, whereas the high short-term mortality feature of ACLF patients was not showed in this study. It is worth trying to do further studies on this ACLF model in order to accumulate more evidence and details.

### 5.2. Immune responses induced ACLF models

Immune responses induced ACLF models are mostly used to imitate autoimmune liver cirrhosis or hepatitis virus induced liver cirrhosis. The occurrence of autoimmune disease mainly due to the dysregulation of immune response, which will result in the breakdown of immune tolerance, and further, lead to the immune mediated organ or tissue damage caused by host itself. The mechanism of autoimmune hepatitis induced liver cirrhosis is still unclear, but there are several hypotheses may account for it. Molecular mimicry is a process that immune system responses to self-components which are similar to external pathogens such as HBV or hepatitis C virus (HCV). Autoantibody like anti-nuclear Antibody (ANA) and smooth muscle antibody (SMA) can be found in these patients, indicating that HBV and HCV may play an important role in autoimmune hepatitis induced liver cirrhosis (99). Besides, genetic factors may also participate in the occurrence of autoimmune hepatitis. Donaldson (100) revealed that major histocompatibility complex (MHC) is associated with autoimmune hepatitis to a large degree. However, no matter what the trigger is, a mass of activated inflammatory cells, especially the CD4 + T helper/inducer cells such as Th1, Th2, and Th17 cells, should be responsible for this immune mediated organ or tissue damage via the secretion of IL-2, IL-6, IFN-γ, and TGF-β.

#### 5.2.1. HSA + D-GalN/LPS

Human serum albumin, a heterologous serum constituent for murine, is usually used to mimic immune response or autoimmune disorder induced chronic liver cirrhosis in rats or fibrosis in mice. The combination of HSA and D-GalN/LPS to establish an ACLF model has a wide application in rats (56, 57, 101, 102). Lots of studies of ACLF are based on this model (57, 101–105). The major limitation of this model is the high mortality during the induction of chronic liver injury and the short survival period after acute hepatic insult like the D-GalN/LPS based models (56). Hu et al. (106) have reported that the mortality of rats during cirrhosis induction was 20% after 2 weeks and 60% after 3 weeks.

#### 5.2.2. PS + D-GalN/LPS

To decrease the mortality during the period of HSA induced liver injury, other heterologous serum such as PS is selected as an alternative because of the low mortality (9).

Acute-on-chronic liver failure models of this combination are established *via* the administration of PS (0.5 ml) twice a week for 11 weeks or 8 weeks intraperitoneally, followed by injection of LPS (50–100 μg/kg) intravenously and D-GalN (600 mg/kg) intraperitoneally (9, 107–109). Recently, Hassan et al. (110) have optimized this combination to develop an ACLF rat model with PS administration (2 ml/kg, twice a week) for 12 consecutive weeks and LPS (100 μg/kg) plus D-GalN (800 mg/kg), demonstrating the classic features of ACLF. ACLF model in this combination has its own advantages in investigating ACLF based on immune mediated chronic liver diseases.

### 5.3. Surgical procedures induced ACLF models

Surgical procedure such as bile duct ligation is appropriate to mimic clinical cases suffer from cholestasis.

#### 5.3.1. BDL + LPS

The combination of surgical procedure with chemical drugs to develop an ideal ACLF model is always an important research direction, and BDL is one of the most commonly used surgery (111). Rats or mice that endure BDL surgery would have an obstructive jaundice to reproduce the hyperbilirubinemia (64, 65). Cirrhosis or fibrosis is confirmed in these rats or mice with cholestasis. Subsequently, a single dose of LPS (11, 12, 91, 92, 112–117) would make the ACLF model established, and this model is widely used in recent years.

Nevertheless, high mortality in early phase after BDL surgery is frequently occurred because the surgical operation would certainly concurrent with tissue damage and high risk of infection especially in mice. Therefore, modified surgical procedures are created, such as reversible BDL (118) and partial BDL (119).

#### 5.3.2. BDL + hepatic ischemia/reperfusion

The combination of BDL surgery and other operations which would cause liver damage to develop ACLF models is a feasible strategy. Surgical based models with liver injury include partial hepatectomy (PHx), hepatic ischemia/reperfusion, and CLP. There are no reports on the combination of BDL or CLP so far. Hu et al. (106) reported an ACLF model combining BDL and hepatic ischemia/reperfusion surgeries in rats to reflect the characteristics of patients progressed to ACLF after liver resection. A reduced-size hepatic ischemia/reperfusion injury procedure was used in this model (120), as well as partial hepatectomy (106). This ACLF model mimics the pathophysiological process, histological characteristics and surgical treatment process well, however, the surgical procedures are too complicated to perform which would limit its application.

### 5.4. The search for an optimal mouse model for ACLF

According to the current understanding and findings on the mechanism ACLF, the clinical course of ACLF could divide into three major stages, including chronic liver injury, acute hepatic/extrahepatic insult, and the excessive systemic inflammatory response caused by over-reactive immune system especially bacterial infection (14). However, due to the lack of optimal experimental animal model for ACLF, the progress of basic study on ACLF is limping. Though the above-mentioned experimental ACLF models were established, none of them can recapitulate and simulate the whole pathological process of ACLF patients.

**FIGURE 2:**
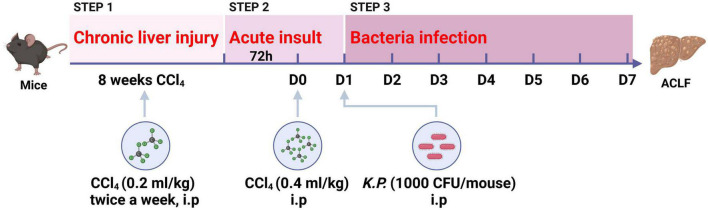
Schematic timeline of the three-step mouse model of acute-on-chronic liver failure (ACLF). Mice were administrated with carbon tetrachloride (CCl_4_) for 8 weeks to induce chronic liver injury, followed by double dosage of CCl_4_ (0.4 ml/kg) injection to induce acute hepatic insult, and *Klebsiella pneumoniae* (*K.P.*) were injected intraperitoneally to induce bacterial infection at 24 h post-acute insult. (Created with BioRender.com).

Recently, we have developed a novel mouse model for ACLF combining chronic liver injury (injection of CCl_4_ for 8 weeks, 0.2 ml/kg), acute hepatic insult (injection of a double dose CCl_4_, 0.4 ml/kg), and bacterial infection (intraperitoneal injection of a single dose *K.P.*, 1,000 CFU/mouse) (Figure 2), recapitulating the major clinical features of patients with ACLF worsened by bacterial infection (14). Moreover, this ACLF model includes chronic liver injury, acute hepatic insult, bacterial infection, renal injury, high short-term mortality, which could simulate the major pathological course of ACLF patients (14). To our knowledge, we introduced for the first time an easy double dose of CCl_4_ injection as acute hepatic insult and a single dose of viable *K.P.* injection to mimic bacterial infection that occurred in most ACLF patients. In addition, systemic inflammatory responses induced by both PAMPs and DAMPs were fully simulated in this model. Importantly, the survival period of this ACLF model has been prolonged to 5–7 days after acute insult, which provide appropriate time for preclinical interventional researches, such as drug screening.

The establish process of this ACLF model was not go well in the beginning. In brief, we first combined CCl_4_ injection with CLP surgery to test if an ACLF model could be developed. Repeated CCl_4_ injection combined with CLP surgery could generate a model with high short-term mortality and sepsis like symptoms, however, no ALT or AST elevation was found even the mice died. Thus, when a double dose of CCl_4_ was added as the acute hepatic insult in the chronic CCl_4_ treated mice, followed by CLP surgery, an ideal ACLF model was established with the three major stages, including chronic liver injury (0.2 ml/kg, CCl_4_ injection twice a week), acute hepatic (a double dose of CCl_4_ injection) insult and polymicrobial infection (CLP surgery) (14). However, the defects of CLP-based ACLF model are obvious. First, it is hard to accurately control the spillage of cecal contents into the peritoneal cavity. Second, the surgical wounding has influence on the pathogenesis of the end stage liver failure. Third, the CLP surgical procedure is more time consuming to some extent.

To improve the CLP-based ACLF model, CS injection was adopted for the substitution of CLP induced polymicrobial infection. We prepared the CS solution according to a recently published protocol and administrated a suggested high dose of 200 μl/mouse in mice (121). However, no mortality was observed in mice injected with CS though the blood cultures showed positive results of bacteriotoxemia (122).

Subsequently, we turned to use single bacterial infection to replace the CLP or CS induced polymicrobial infection. Clinically, *Escherichia coli* (*E. Coli*) and *K.P.* are in the top rank of pathogens in ACLF patients (123). Different doses of *E. Coli* were first sent to the test. Surprisingly, mortality in mice was able to be observed after *E. Coli* injection till at a dose of 10^8^ CFU/mouse (14), which is too high to apply. Subsequently, different doses of *K.P.* were tested and an optimal mortality with appropriate survival period was found at a dose of 1,000 CFU/mouse (14). Meanwhile, *Salmonella* at a dose of 8,000 CFU/mouse was found similar results like *K.P.* (84). Therefore, a three-step ACLF model has been developed in mice, which could not only recapitulate the major three stages of ACLF, but also prolong the animal survival period with longer observation and interventional time for screening drugs and mechanism studies (Figure 2).

## 6. Prospect and conclusion

The efforts for developing an optimal animal model for the study of ACLF are far from over. Along with the further understanding for pathophysiological mechanism of ACLF, more precise and perfect animal models would be established in the near future. To date, there are three main patterns of ACLF experimental models worldwide, which were induced via hepatotoxic reagents, immune responses, and surgical procedures, respectively. Generally, each pattern of ACLF model always reflects a particular aspect of ACLF patients clinically, and it is very difficult to construct a single model to meet all the aspects for the study of ACLF. Similar like the controversy in the definitions and criteria of ACLF among APASL, ESAL, and AASLD, animal models for ACLF would not be consistent till there is a universal agreement on the mechanism and definition of ACLF globally. At present, on the way to further reveal and elucidate the pathogenesis of ACLF, the optimal animal model of ACLF should be selected by the purpose of the study.

## Author contributions

XX, HZ, and JZ wrote the manuscript. XX, DS, and CZ reviewed and revised the manuscript. All authors contributed to the article and approved the submitted version.

## References

[1] Moreau R Jalan R Gines P Pavesi M Angeli P Cordoba J Acute-on-chronic liver failure is a distinct syndrome that develops in patients with acute decompensation of cirrhosis. *Gastroenterology.* (2013) 144:1437.e1–9. 10.1053/j.gastro.2013.02.042 23474284

[2] Mahmud N Kaplan D Taddei T Goldberg D. Incidence and mortality of acute-on-chronic liver failure using two definitions in patients with compensated cirrhosis. *Hepatology.* (2019) 69:2150–63. 30615211 10.1002/hep.30494 PMC6461492

[3] Khanam A Kottilil S. Acute-on-chronic liver failure: pathophysiological mechanisms and management. *Front Med.* (2021) 8:752875. 10.3389/fmed.2021.752875 34820395 PMC8606418

[4] Arroyo V Moreau R Kamath P Jalan R Gines P Nevens F Acute-on-chronic liver failure in cirrhosis. *Nat Rev Dis Primers.* (2016) 2:16041. 27277335 10.1038/nrdp.2016.41

[5] Bajaj J O’Leary J Lai J Wong F Long M Wong R Acute-on-chronic liver failure clinical guidelines. *Am J Gastroenterol.* (2022) 117:225–52. 35006099 10.14309/ajg.0000000000001595

[6] Wu T Li J Shao L Xin J Jiang L Zhou Q Development of diagnostic criteria and a prognostic score for hepatitis B virus-related acute-on-chronic liver failure. *Gut.* (2018) 67:2181–91. 10.1136/gutjnl-2017-314641 28928275

[7] Cao Z Liu Y Wang S Lu X Yin S Jiang S The impact of HBV flare on the outcome of HBV-related decompensated cirrhosis patients with bacterial infection. *Liver Int.* (2019) 39:1943–53. 31206235 10.1111/liv.14176

[8] Moreau R. The Pathogenesis of ACLF: the inflammatory response and immune function. *Semin Liver Dis.* (2016) 36:133–40. 27172355 10.1055/s-0036-1583199

[9] Li F Miao L Sun H Zhang Y Bao X Zhang D. Establishment of a new acute-on-chronic liver failure model. *Acta Pharm Sin B.* (2017) 7:326–33. 28540169 10.1016/j.apsb.2016.09.003 PMC5430813

[10] Li X Wang L Wang L Han X Yang F Gong Z. Blockade of high-mobility group box-1 ameliorates acute on chronic liver failure in rats. *Inflamm Res.* (2013) 62:703–9. 10.1007/s00011-013-0624-1 23591781

[11] Balasubramaniyan V Dhar D Warner A Vivien LW Amiri A Bright B. Importance of Connexin-43 based gap junction in cirrhosis and acute-on-chronic liver failure. *J Hepatol.* (2013) 58:1194–200. 10.1016/j.jhep.2013.01.023 23376361

[12] Tripathi D Vilaseca M Lafoz E Garcia-Caldero H Viegas Haute G Fernandez-Iglesias A Simvastatin prevents progression of acute on chronic liver failure in rats with cirrhosis and portal hypertension. *Gastroenterology.* (2018) 155:1564–77. 10.1053/j.gastro.2018.07.022 30055171

[13] Kuhla A Eipel C Abshagen K Siebert N Menger M Vollmar B. Role of the perforin/granzyme cell death pathway in D-Gal/LPS-induced inflammatory liver injury. *Am J Physiol Gastrointest Liver Physiol.* (2009) 296:G1069–76. 10.1152/ajpgi.90689.2008 19264954

[14] Xiang X Feng D Hwang S Ren T Wang X Trojnar E Interleukin-22 ameliorates acute-on-chronic liver failure by reprogramming impaired regeneration pathways in mice. *J Hepatol.* (2020) 72:736–45. 10.1016/j.jhep.2019.11.013 31786256 PMC7085428

[15] Arroyo V Moreau R Jalan R. Acute-on-chronic liver failure. *N Engl J Med.* (2020) 382:2137–45. 32459924 10.1056/NEJMra1914900

[16] Ginés P Quintero E Arroyo V Terés J Bruguera M Rimola A Compensated cirrhosis: natural history and prognostic factors. *Hepatology.* (1987) 7:122–8. 3804191 10.1002/hep.1840070124

[17] Takeuchi O Akira S. Pattern recognition receptors and inflammation. *Cell.* (2010) 140:805–20. 20303872 10.1016/j.cell.2010.01.022

[18] Kono H Rock K. How dying cells alert the immune system to danger. *Nat Rev Immunol.* (2008) 8:279–89. 18340345 10.1038/nri2215 PMC2763408

[19] Louvet A Wartel F Castel H Dharancy S Hollebecque A Canva-Delcambre V Infection in patients with severe alcoholic hepatitis treated with steroids: early response to therapy is the key factor. *Gastroenterology.* (2009) 137:541–8. 19445945 10.1053/j.gastro.2009.04.062

[20] Rajiv Jalan V. Role of predisposition, injury, response and organ failure in the prognosis of patients with acute-onchronic liver failure: a prospective cohort study. *Crit Care.* (2012) 16:R227. 10.1186/cc11882 23186071 PMC3672612

[21] Bernardi M Moreau R Angeli P Schnabl B Arroyo V. Mechanisms of decompensation and organ failure in cirrhosis: from peripheral arterial vasodilation to systemic inflammation hypothesis. *J Hepatol.* (2015) 63:1272–84. 26192220 10.1016/j.jhep.2015.07.004

[22] Clària J Stauber R Coenraad M Moreau R Jalan R Pavesi M Systemic inflammation in decompensated cirrhosis: characterization and role in acute-on-chronic liver failure. *Hepatology.* (2016) 64:1249–64. 27483394 10.1002/hep.28740

[23] Arroyo V Angeli P Moreau R Jalan R Clària J Trebicka J The systemic inflammation hypothesis: towards a new paradigm of acute decompensation and multiorgan failure in cirrhosis. *J Hepatol.* (2021) 74:670–85. 10.1016/j.jhep.2020.11.048 33301825

[24] Kim H Chang Y Park J Ahn H Cho H Han S Characterization of acute-on-chronic liver failure and prediction of mortality in Asian patients with active alcoholism. *J Gastroenterol Hepatol.* (2016) 31:427–33. 10.1111/jgh.13084 26260091

[25] Suntharalingam G Perry M Ward S Brett S Castello-Cortes A Brunner M Cytokine storm in a phase 1 trial of the anti-CD28 monoclonal antibody TGN1412. *N Engl J Med.* (2006) 355:1018–28. 10.1056/NEJMoa063842 16908486

[26] Casulleras M Zhang I Lopez-Vicario C Claria J. Leukocytes, systemic inflammation and immunopathology in acute-on-chronic liver failure. *Cells.* (2020) 9:2632. 33302342 10.3390/cells9122632 PMC7762372

[27] Khanam A Kottilil S. Abnormal innate immunity in acute-on-chronic liver failure: immunotargets for therapeutics. *Front Immunol.* (2020) 11:2013. 10.3389/fimmu.2020.02013 33117329 PMC7578249

[28] Trebicka J Amoros A Pitarch C Titos E Alcaraz-Quiles J Schierwagen R Addressing profiles of systemic inflammation across the different clinical phenotypes of acutely decompensated cirrhosis. *Front Immunol.* (2019) 10:476. 10.3389/fimmu.2019.00476 30941129 PMC6434999

[29] Zaccherini G Weiss E Moreau R. Acute-on-chronic liver failure: definitions, pathophysiology and principles of treatment. *JHEP Rep.* (2021) 3:100176. 33205036 10.1016/j.jhepr.2020.100176 PMC7652714

[30] Lange C Moreau R. Immunodysfunction in acute-on-chronic liver failure. *Visc Med.* (2018) 34:276–82. 30345285 10.1159/000488690 PMC6189545

[31] Bernsmeier C Pop O Singanayagam A Triantafyllou E Patel V Weston C Patients with acute-on-chronic liver failure have increased numbers of regulatory immune cells expressing the receptor tyrosine kinase MERTK. *Gastroenterology.* (2015) 148:603–15.e14. 10.1053/j.gastro.2014.11.045 25479139

[32] Bernsmeier C Triantafyllou E Brenig R Lebosse F Singanayagam A Patel V CD14(+) CD15(-) HLA-DR(-) myeloid-derived suppressor cells impair antimicrobial responses in patients with acute-on-chronic liver failure. *Gut.* (2018) 67:1155–67. 10.1136/gutjnl-2017-314184 28592438 PMC5969362

[33] O’Brien A Fullerton J Massey K Auld G Sewell G James S Immunosuppression in acutely decompensated cirrhosis is mediated by prostaglandin E2. *Nat Med.* (2014) 20:518–23. 24728410 10.1038/nm.3516 PMC5369639

[34] Jimenez W. Carbon tetrachloride induced cirrhosis in rats: a useful tool for investigating the pathogenesis of ascites in chronic liver disease. *J Gastroenterol Heparol.* (1992) 7:90–7. 10.1111/j.1440-1746.1992.tb00940.x 1543874

[35] Jang J Kang K Kim Y Kang Y Lee I. Reevaluation of experimental model of hepatic fibrosis induced by hepatotoxic drugs: an easy, applicable, and reproducible model. *Transplant Proc.* (2008) 40:2700–3. 10.1016/j.transproceed.2008.07.040 18929839

[36] Pérez Tamayo R. Is cirrhosis of the liver experimentally produced by CCl4 and adequate model of human cirrhosis? *Hepatology.* (1983) 3:112–20. 10.1002/hep.1840030118 6337081

[37] Boll M. Mechanism of carbon tetrachloride-induced hepatotoxicity. hepatocellular damage by reactive carbon tetrachloride metabolites. *Z Naturforsch.* (2001) 56c:649–59. 10.1515/znc-2001-7-826 11531102

[38] McLean E McLean A Sutton P. Instant cirrhosis. An improved method for producing cirrhosis of the liver in rats by simultaneous administration of carbon tetrachloride and phenobarbitone. *Br J Exp Pathol.* (1969) 50:502–6. 5388497 PMC2072144

[39] Seyer J. Interstitial collagen polymorphism in rat liver with CCl4-induced cirrhosis. *Biochim Biophys Acta.* (1980) 629:490–8. 10.1016/0304-4165(80)90154-3 7417507

[40] Vorobioff J Bredfeldt J Groszmann R. Increased blood flow through the portal system in cirrhotic rats. *Gastroenterology.* (1984) 87:1120–6. 6479534

[41] Schuppan D Dumont J Kim K Hennings G Hahn E. Serum concentration of the aminoterminal procollagen type III peptide in the rat reflects early formation of connective tissue in experimental liver cirrhosis. *J Hepatol.* (1986) 3:27–37. 10.1016/s0168-8278(86)80142-8 3745883

[42] Proctor E Chatamra K. High yield micronodular cirrhosis in the rat. *Gastroenterology.* (1982) 83:1183–90. 7129027

[43] López-Novoa J Rengel M Hernando L. Dynamics of ascites formation in rats with experimental cirrhosis. *Am J Physiol.* (1980) 238:F353–7. 7377346 10.1152/ajprenal.1980.238.5.F353

[44] Silverstein R. D-galactosamine lethality model: scope and limitations. *J Endotoxin Res.* (2004) 10:147–62. 10.1179/096805104225004879 15198850

[45] Low T Leow C Salto-Tellez M Chung MC. A proteomic analysis of thioacetamide-induced hepatotoxicity and cirrhosis in rat livers. *Proteomics.* (2004) 4:3960–74. 10.1002/pmic.200400852 15526343

[46] Dwivedi D Jena G. Glibenclamide protects against thioacetamide-induced hepatic damage in Wistar rat: investigation on NLRP3, MMP-2, and stellate cell activation. *Naunyn Schmiedebergs Arch Pharmacol.* (2018) 391:1257–74. 10.1007/s00210-018-1540-2 30066023

[47] Lee S Kim S Min S Kim K. Ideal experimental rat models for liver diseases. *Korean J Hepatobiliary Pancreat Surg.* (2011) 15:67–77. 26421020 10.14701/kjhbps.2011.15.2.67 PMC4582547

[48] Larsen F Wendon J. Understanding paracetamol-induced liver failure. *Intensive Care Med.* (2014) 40:888–90. 24737263 10.1007/s00134-014-3293-9

[49] Mossanen J Tacke F. Acetaminophen-induced acute liver injury in mice. *Lab Anim.* (2015) 49(Suppl. 1):30–6. 10.1177/0023677215570992 25835736

[50] Kubes P Mehal W. Sterile inflammation in the liver. *Gastroenterology.* (2012) 143:1158–72. 22982943 10.1053/j.gastro.2012.09.008

[51] Wang J Cunningham B Edelman G. Unusual fragments in the subunit structure of concanavalin A. *Proc Natl Acad Sci USA.* (1971) 68:1130–4. 5288363 10.1073/pnas.68.6.1130 PMC389135

[52] Dwyer J Johnson C. The use of concanavalin A to study the immunoregulation of human T cells. *Clin Exp Immunol.* (1981) 46:237–49. 6461456 PMC1536405

[53] Liu Y Hao H Hou T. Concanavalin A-induced autoimmune hepatitis model in mice: mechanisms and future outlook. *Open Life Sci.* (2022) 17:91–101. 10.1515/biol-2022-0013 35291566 PMC8886606

[54] Merlot A Kalinowski D Richardson D. Unraveling the mysteries of serum albumin-more than just a serum protein. *Front Physiol.* (2014) 5:299. 10.3389/fphys.2014.00299 25161624 PMC4129365

[55] Paronetto F Popper H. Chronic liver injury induced by immunologic reactions. Cirrhosis following immunization with heterologous sera. *Am J Pathol.* (1966) 49:1087–101. 5297057 PMC1907285

[56] Dong Z Liu J Shen H Ma H Jia J. [Immune complex induced rat liver fibrosis model by intraperitoneal injection of human serum albumin]. *Zhonghua Shi Yan He Lin Chuang Bing Du Xue Za Zhi.* (2006) 20:12–5. 16642209

[57] Yang F Li X Wang L Wang L Han X Zhang H Inhibitions of NF-kappaB and TNF-alpha result in differential effects in rats with acute on chronic liver failure induced by d-Gal and LPS. *Inflammation.* (2014) 37:848–57. 10.1007/s10753-013-9805-x 24385241

[58] Bhunchet E. Contribution of immune response to the hepatic fibrosis induced by porcine serum. *Hepatology.* (1996) 23:811–7. 10.1053/jhep.1996.v23.pm0008666336 8666336

[59] Baba Y Uetsuka K Nakayama H Dot K. Rat strain differences in the early stage of porcine-serum-induced hepatic fibrosis. *Exp Toxicol Pathol.* (2004) 55:325–30. 10.1078/0940-2993-00336 15088634

[60] Tsukamoto H Matsuoka M French S. Experimental models of hepatic fibrosis: a review. *Semin Liver Dis.* (1990) 10:56–65. 2110685 10.1055/s-2008-1040457

[61] Schuppan D Ruehl M Somasundaram R Hahn E. Matrix as a modulator of hepatic fibrogenesis. *Semin Liver Dis.* (2001) 21:351–72. 11586465 10.1055/s-2001-17556

[62] Villeneuve J. The natural history of chronic hepatitis B virus infection. *J Clin Virol.* (2005) 34(Suppl. 1):S139–42. 16461215 10.1016/s1386-6532(05)80024-1

[63] Cameron G Oakley C. Ligation of the common bile duct. *J Pathol Bacteriol.* (1932) 35:769–98.

[64] Kountouras J Billing B Scheuer P. Prolonged bile duct obstruction: a new experimental model for cirrhosis in the rat. *Br J Exp Pathol.* (1984) 65:305–11. 6743531 PMC2040968

[65] Varga Z Erdelyi K Paloczi J Cinar R Zsengeller Z Jourdan T Disruption of renal arginine metabolism promotes kidney injury in hepatorenal syndrome in mice. *Hepatology.* (2018) 68:1519–33. 10.1002/hep.29915 29631342 PMC6173643

[66] Colares J Schemitt E Hartmann R Licks F Soares M Bosco A Antioxidant and anti-inflammatory action of melatonin in an experimental model of secondary biliary cirrhosis induced by bile duct ligation. *World J Gastroenterol.* (2016) 22:8918–28. 10.3748/wjg.v22.i40.8918 27833383 PMC5083797

[67] Zhang S Li T Soyama A Tanaka T Yan C Sakai Y Up-regulated extracellular matrix components and inflammatory chemokines may impair the regeneration of cholestatic liver. *Sci Rep.* (2016) 6:26540. 10.1038/srep26540 27226149 PMC4880910

[68] Zepeda-Morales A Del Toro-Arreola S García-Benavides L Bastidas-Ramírez B Fafutis-Morris M Pereira-Suárez A. Liver fibrosis in bile duct-ligated rats correlates with increased hepatic IL-17 and TGF-β2 expression. *Ann Hepatol.* (2016) 15:418–26. 10.5604/16652681.1198820 27049496

[69] Raetz C Whitfield C. Lipopolysaccharide endotoxins. *Annu Rev Biochem.* (2002) 71:635–700. 12045108 10.1146/annurev.biochem.71.110601.135414 PMC2569852

[70] Bertani B Ruiz N. Function and biogenesis of lipopolysaccharides. *EcoSal Plus.* (2018) 8. 10.1128/ecosalplus.ESP-0001-2018 30066669 PMC6091223

[71] Beutler B Rietschel E. Innate immune sensing and its roots: the story of endotoxin. *Nat Rev Immunol.* (2003) 3:169–76. 10.1038/nri1004 12563300

[72] Rathinam V Zhao Y Shao F. Innate immunity to intracellular LPS. *Nat Immunol.* (2019) 20:527–33. 30962589 10.1038/s41590-019-0368-3 PMC7668400

[73] Good D George T Watts B III. Toll-like receptor 2 is required for LPS-induced Toll-like receptor 4 signaling and inhibition of ion transport in renal thick ascending limb. *J Biol Chem.* (2012) 287:20208–20. 10.1074/jbc.M111.336255 22523073 PMC3370203

[74] Hamesch K Borkham-Kamphorst E Strnad P Weiskirchen R. Lipopolysaccharide-induced inflammatory liver injury in mice. *Lab Anim.* (2015) 49(Suppl. 1):37–46. 25835737 10.1177/0023677215570087

[75] Rahman T Hodgson H. Animal models of acute hepatic failure. *Int J Exp Pathol.* (2000) 81:145–57. 10762442 10.1046/j.1365-2613.2000.00144.x PMC2517718

[76] Jirillo E Caccavo D Magrone T Piccigallo E Amati L Lembo A The role of the liver in the response to LPS: experimental and clinical findings. *J Endotoxin Res.* (2002) 8:319–27. 12537690 10.1179/096805102125000641

[77] Rittirsch D Hoesel L Ward P. The disconnect between animal models of sepsis and human sepsis. *J Leukoc Biol.* (2007) 81:137–43. 17020929 10.1189/jlb.0806542

[78] Wichterman K Baue A Chaudry I. Sepsis and septic shock–a review of laboratory models and a proposal. *J Surg Res.* (1980) 29:189–201. 10.1016/0022-4804(80)90037-2 6997619

[79] Rittirsch D Huber-Lang M Flierl M Ward P. Immunodesign of experimental sepsis by cecal ligation and puncture. *Nat Protoc.* (2009) 4:31–6. 10.1038/nprot.2008.214 19131954 PMC2754226

[80] Rincon J Efron P Moldawer L Larson S. Cecal slurry injection in neonatal and adult mice. *Methods Mol Biol.* (2021) 2321:27–41. 34048005 10.1007/978-1-0716-1488-4_4 PMC8482797

[81] Wynn J Scumpia P Delano M O’Malley K Ungaro R Abouhamze A Increased mortality and altered immunity in neonatal sepsis produced by generalized peritonitis. *Shock.* (2007) 28:675–83. 10.1097/SHK.0b013e3180556d09 17621256

[82] Xu M Feng D Wu H Wang H Chan Y Kolls J Liver is the major source of elevated serum lipocalin-2 levels after bacterial infection or partial hepatectomy: a critical role for IL-6/STAT3. *Hepatology.* (2015) 61:692–702. 25234944 10.1002/hep.27447 PMC4303493

[83] Zheng M Horne W McAleer J Pociask D Eddens T Good M Therapeutic Role of Interleukin 22 in Experimental Intra-abdominal *Klebsiella pneumoniae* Infection in Mice. *Infect Immun.* (2016) 84:782–9. 10.1128/IAI.01268-15 26729763 PMC4771339

[84] Zhang J Zhai H Yu P Shang D Mo R Li Z Human umbilical cord blood mononuclear cells ameliorate CCl4-induced acute liver injury in mice via inhibiting inflammatory responses and upregulating peripheral interleukin-22. *Front Pharmacol.* (2022) 13:924464. 10.3389/fphar.2022.924464 35942221 PMC9356225

[85] Cai Y Kimura S. Noninvasive intratracheal intubation to study the pathology and physiology of mouse lung. *J Vis Exp.* (2013) 8:e50601. 10.3791/50601 24300823 PMC3984658

[86] Zhang Y Chen X Sun D. Effects of coencapsulation of hepatocytes with adipose-derived stem cells in the treatment of rats with acute-on-chronic liver failure. *Int J Artif Organs.* (2014) 37:133–41. 10.5301/ijao.5000284 24619896 PMC6161594

[87] Diao J. SHYCD induces APE1/Ref-1 subcellular localization to regulate the p53-apoptosis signaling pathway in the prevention and treatment of acute on chronic liver failure. *Oncotarget.* (2017) 8:84782–97. 10.18632/oncotarget.19891 29156683 PMC5689573

[88] Fortea J Fernández-Mena C Puerto M Ripoll C Almagro J Bañares J Comparison of two protocols of carbon tetrachloride-induced cirrhosis in rats - improving yield and reproducibility. *Sci Rep.* (2018) 8:9163. 10.1038/s41598-018-27427-9 29907790 PMC6003930

[89] Hou W Wei X Liang J Fang P Ma C Zhang Q HMGB1-induced hepatocyte pyroptosis expanding inflammatory responses contributes to the pathogenesis of acute-on-chronic liver failure (ACLF). *J Inflamm Res.* (2021) 14:7295–313. 10.2147/JIR.S336626 34992418 PMC8711847

[90] De Minicis S Seki E Uchinami H Kluwe J Zhang Y Brenner D Gene expression profiles during hepatic stellate cell activation in culture and in vivo. *Gastroenterology.* (2007) 132:1937–46. 17484886 10.1053/j.gastro.2007.02.033

[91] Engelmann C Sheikh M Sharma S Kondo T Loeffler-Wirth H Zheng Y Toll-like receptor 4 is a therapeutic target for prevention and treatment of liver failure. *J Hepatol.* (2020) 73:102–12. 31987990 10.1016/j.jhep.2020.01.011

[92] Kondo T Macdonald S Engelmann C Habtesion A Macnaughtan J Mehta G The role of RIPK1 mediated cell death in acute on chronic liver failure. *Cell Death Dis.* (2021) 13:5. 34921136 10.1038/s41419-021-04442-9 PMC8683430

[93] Zhang J Gao J Lin D Xiong J Wang J Chen J Potential Networks Regulated by MSCs in Acute-On-Chronic Liver Failure: exosomal miRNAs and Intracellular Target Genes. *Front Genet.* (2021) 12:650536. 10.3389/fgene.2021.650536 33968135 PMC8102832

[94] Bai L Kong M Duan Z Liu S Zheng S Chen Y. M2-like macrophages exert hepatoprotection in acute-on-chronic liver failure through inhibiting necroptosis-S100A9-necroinflammation axis. *Cell Death Dis.* (2021) 12:93. 10.1038/s41419-020-03378-w 33462187 PMC7814003

[95] Ni S Li S Yang N Tang X Zhang S Hu D Deregulation of Regulatory T Cells in Acute-on-Chronic Liver Failure: a Rat Model. *Mediators Inflamm.* (2017) 2017:1390458. 10.1155/2017/1390458 28194045 PMC5282067

[96] Cerini F Vilaseca M Lafoz E García-Irigoyen O García-Calderó H Tripathi D Enoxaparin reduces hepatic vascular resistance and portal pressure in cirrhotic rats. *J Hepatol.* (2016) 64:834–42. 10.1016/j.jhep.2015.12.003 26686269

[97] de Mesquita F Guixe-Muntet S Fernandez-Iglesias A Maeso-Diaz R Vila S Hide D Liraglutide improves liver microvascular dysfunction in cirrhosis: evidence from translational studies. *Sci Rep.* (2017) 7:3255. 10.1038/s41598-017-02866-y 28607430 PMC5468330

[98] Nautiyal N Maheshwari D Tripathi D Kumar D Kumari R Gupta S Establishment of a murine model of acute-on-chronic liver failure with multi-organ dysfunction. *Hepatol Int.* (2021) 15:1389–401. 10.1007/s12072-021-10244-0 34435344

[99] Liberal R Longhi M Mieli-Vergani G Vergani D. Pathogenesis of autoimmune hepatitis. *Best Pract Res Clin Gastroenterol.* (2011) 25:653–64. 22117632 10.1016/j.bpg.2011.09.009

[100] Donaldson P. Genetics of liver disease: immunogenetics and disease pathogenesis. *Gut.* (2004) 53:599–608. 15016758 10.1136/gut.2003.031732 PMC1773998

[101] Wang L Wang L Chen H Fan C Li X He C Ethyl pyruvate protects against experimental acute-on-chronic liver failure in rats. *World J Gastroenterol.* (2012) 18:5709–18. 10.3748/wjg.v18.i40.5709 23155311 PMC3484339

[102] Liu X Chen Y Wang T Lu J Zhang L Song C [Establishment of a D-galactosamine/lipopolysaccharide induced acute-on-chronic liver failure model in rats]. *Zhonghua Gan Zang Bing Za Zhi.* (2007) 15:771–5. 17963606

[103] Xu Y Wang H Bao S Tabassam F Cai W Xiang X Amelioration of liver injury by continuously targeted intervention against TNFRp55 in rats with acute-on-chronic liver failure. *PLoS One.* (2013) 8:e68757. 10.1371/journal.pone.0068757 23874752 PMC3712937

[104] Gao D Fu J Qin B Huang W Yang C Jia B. Recombinant adenovirus containing hyper-interleukin-6 and hepatocyte growth factor ameliorates acute-on-chronic liver failure in rats. *World J Gastroenterol.* (2016) 22:4136–48. 10.3748/wjg.v22.i16.4136 27122664 PMC4837431

[105] Hou W Hao Y Yang W Tian T Fang P Du Y The Jieduan-Niwan (JDNW) formula ameliorates hepatocyte apoptosis: a study of the inhibition of E2F1-mediated apoptosis signaling pathways in acute-on-chronic liver failure (ACLF) Using Rats. *Drug Des Devel Ther.* (2021) 15:3845–62. 10.2147/DDDT.S308713 34526765 PMC8436178

[106] Hu C Shen S Zhang A Ren B Lin F. The liver protective effect of methylprednisolone on a new experimental acute-on-chronic liver failure model in rats. *Dig Liver Dis.* (2014) 46:928–35. 10.1016/j.dld.2014.06.008 25022338

[107] Wang S Li M Miao L Wu S Tong Y Zhang W Protective effects of a novel water-soluble biphenyl compound WLP-S-14 against acute-on-chronic liver failure in rats. *J Asian Nat Prod Res.* (2019) 21:928–38. 10.1080/10286020.2019.1585822 31111726

[108] Li F Liu N Liu W Li M Zhang F Dong Z Role of dihydroceramides in the progression of acute-on-chronic liver failure in rats. *Chin Med J.* (2020) 133:198–204. 10.1097/CM9.0000000000000601 31880746 PMC7028171

[109] Li J Zhang Q Gao L Du Y Chen Y. Efficacy of decoction from Jieduan Niwan formula on rat model of acute-on-chronic liver failure induced by porcine serum. *J Tradit Chin Med.* (2020) 40:602–12. 10.19852/j.cnki.jtcm.2020.04.009 32744027

[110] Hassan H Cai Q Liang X Xin J Ren K Jiang J Transcriptomics reveals immune-metabolism disorder in acute-on-chronic liver failure in rats. *Life Sci Alliance.* (2022) 5:e202101189. 10.26508/lsa.202101189 34853163 PMC8645333

[111] Harry D. Increased sensitivity to endotoxemia in the bile duct–ligated cirrhotic rat. *Hepatology.* (1999) 30:1198–205. 10.1002/hep.510300515 10534341

[112] Wright G Davies N Shawcross D Hodges S Zwingmann C Brooks H Endotoxemia produces coma and brain swelling in bile duct ligated rats. *Hepatology.* (2007) 45:1517–26. 10.1002/hep.21599 17523148

[113] Shah N Dhar D El Zahraa Mohammed F Habtesion A Davies N Jover-Cobos M. Prevention of acute kidney injury in a rodent model of cirrhosis following selective gut decontamination is associated with reduced renal TLR4 expression. *J Hepatol.* (2012) 56:1047–53. 10.1016/j.jhep.2011.11.024 22266601

[114] Engelmann C Adebayo D Oria M De Chiara F Novelli S Habtesion A Recombinant alkaline phosphatase prevents acute on chronic liver failure. *Sci Rep.* (2020) 10:389. 10.1038/s41598-019-57284-z 31942020 PMC6962206

[115] Queck A Bode H Uschner F Brol M Graf C Schulz M Systemic MCP-1 levels derive mainly from injured liver and are associated with complications in cirrhosis. *Front Immunol.* (2020) 11:354. 10.3389/fimmu.2020.00354 32218781 PMC7078155

[116] Chouhan M Taylor S Bainbridge A Walker-Samuel S Davies N Halligan S Haemodynamic changes in cirrhosis following terlipressin and induction of sepsis-a preclinical study using caval subtraction phase-contrast and cardiac MRI. *Eur Radiol.* (2021) 31:2518–28. 10.1007/s00330-020-07259-w 33044649 PMC7979649

[117] Monteiro S Grandt J Uschner F Kimer N Madsen J Schierwagen R Differential inflammasome activation predisposes to acute-on-chronic liver failure in human and experimental cirrhosis with and without previous decompensation. *Gut.* (2021) 70:379–87. 10.1136/gutjnl-2019-320170 32241903 PMC7815638

[118] Raven A Lu W Man T Ferreira-Gonzalez S O’Duibhir E Dwyer B Cholangiocytes act as facultative liver stem cells during impaired hepatocyte regeneration. *Nature.* (2017) 547:350–4. 28700576 10.1038/nature23015 PMC5522613

[119] Aller M Arias N Prieto I Agudo S Gilsanz C Lorente L A half century (1961-2011) of applying microsurgery to experimental liver research. *World J Hepatol.* (2012) 4:199–208. 10.4254/wjh.v4.i7.199 22855695 PMC3409354

[120] Kohli V Madden J Bentley R Clavien P. Calpain mediates ischemic injury of the liver through modulation of apoptosis and necrosis. *Gastroenterology.* (1999) 116:168–78. 10.1016/s0016-5085(99)70241-6 9869615

[121] Starr M Steele A Saito M Hacker B Evers B Saito H. A new cecal slurry preparation protocol with improved long-term reproducibility for animal models of sepsis. *PLoS One.* (2014) 9:e115705. 10.1371/journal.pone.0115705 25531402 PMC4274114

[122] Xiang X Hwang S Gao B. Reply to: “Interleukin-22 in acute-on-chronic liver failure: a matter of ineffective levels, receptor dysregulation or defective signalling?”: the search for an optimal mouse model. *J Hepatol.* (2020) 73:982–4. 32690377 10.1016/j.jhep.2020.06.002

[123] Bajaj J Kamath P Reddy K. The evolving challenge of infections in cirrhosis. *N Engl J Med.* (2021) 384:2317–30. 34133861 10.1056/NEJMra2021808

